# Editorial: “Unravelling neural stem cell biology: players and strategies”

**DOI:** 10.3389/fcell.2023.1206339

**Published:** 2023-05-02

**Authors:** Ma Salomé Sirerol-Piquer, Jose Manuel Morante-Redolat, Eva Porlan

**Affiliations:** ^1^ Centro de Investigación Biomédica en Red de Enfermedades Neurodegenerativas (CIBERNED), Madrid, Spain; ^2^ Departamento de Biología Celular, Biología Funcional y Antropología Física, Universidad de Valencia, Valencia, Spain; ^3^ Instituto de Biotecnología y Biomedicina (BioTecMed), Universidad de Valencia, Valencia, Spain; ^4^ Departamento de Biología Molecular, Universidad Autónoma de Madrid (UAM), Madrid, Spain; ^5^ Centro de Biología Molecular “Severo Ochoa” (CSIC-UAM), Madrid, Spain; ^6^ Instituto Universitario de Biología Molecular—UAM, Madrid, Spain; ^7^ Instituto de Investigación Sanitaria del Hospital Universitario La Paz (IdiPAZ), Instituto de Salud Carlos III, Madrid, Spain

**Keywords:** neural stem cells, adult neurogenesis, quiescence, ventricular-subventricular zone, dentate gyrus, neurogenic niche, regulating factors

The properties and behaviour of adult or somatic neural stem cells (NSCs) are governed by a complex network of intrinsic molecules and pathways along with a milieu of extrinsic signals hailed from the extremely controlled environment or niche in which they reside. These NSCs are able to self-sustain while dividing to produce highly proliferative intermediate progenitors that, in turn, give rise to neuroblasts and neurons. Plasticity in the adult brain is mainly fostered by the generation of new neurons and their functional integration, a process that remains far from being well understood. This phenomenon has been shown to occur in vertebrates: fish, amphibians, reptiles, birds and mammals, with conflicting results regarding humans, leading to a yet unresolved debate. However, a consensus exists over the notion that the extent and functional relevance of adult neurogenesis is different throughout vertebrate classes (Kempermann, 2015).

In mammals, adult neurogenesis occurs mainly in the olfactory bulbs and the dentate gyrus of the hippocampus, with neurons arising from lateral ventricle walls- or dentate gyrus-residing NSCs, respectively. Neurogenesis beyond these *canonical* subependymal (also known as ventricular-subventricular zone or V-SVZ) and hippocampal niches has also been reported but it remains deeply controversial (Leal-Galicia et al., 2021). In this special number, Fernández Acosta et al. follow up previous work (Defterali et al., 2021) and describe the presence and activity of local NSCs in the murine olfactory bulb core, which contribute with new olfactory neurons distinct from those differentiating from neuroblasts that migrate from the lateral ventricle niche, with differential dendrite arborization depending on their final integration position in the periglomerular or the granular layers.

Transcriptomic and proteomic analyses have partially decoded NSC molecular signatures and have helped to unveil the complexity of the molecular network of signals that rules NSC biology (Kjell et al., 2020; Kalinina and Lagace, 2022). Amongst these, extracellular niche cues stand-out as key instructors of NSC cellular decisions. In their review article, Quaresima et al. thoroughly revise the current knowledge on the structure and the peculiarities of the V-SVZ neurogenic niche. They also discuss the contribution of the extracellular signals provided by the niche components to the network with a special emphasis set on ependymal and vascular components of the niche. They discuss the role of signals produced by endothelial cells and pericytes, as well as the involvement of choroid plexus-derived cerebrospinal fluid cues. Finally, the authors consider the possible implications of the latter in pathological situations such as hydrocephalus.

Single-cell analysis combined with retrospective isolation has allowed for the first time the identification of NSC in three proliferative states that coexist in the neurogenic niche. Quiescence is a strictly regulated property that allows cells to reversibly exit proliferation. This ability protects NSC pools from eventual exhaustion and DNA damage accumulation through adulthood and is currently regarded as one of the hallmarks of stem cell ageing (Kalamakis et al., 2019). To understand the molecular mechanisms ruling NSC entry and exit from quiescence is paramount to fully comprehend the process of adult neurogenesis. Unveiling this process may partly disclose why active and prolific niches correlate inversely with evolutionary complexity and open a window of opportunity for therapeutically manipulating NSC and neurogenesis. In this context, the original article by Garcia-Corzo et al. describe how alternative splicing of Wnt signalling pathway component T cell factor/Lymphoid enhancer factor 1 (TCF/LEF1) gene is a novel post-transcriptional mechanism by which hippocampal NSC maintain quiescence, revealing another layer to the already complex network of signals involved in NSC regulation. Dormancy is a strategy by which cancer cells bypass cytotoxic therapies and drive tumour recurrence, underscoring the role of quiescence in oncology. Moreover, NSC from the subependymal niche have been hypothesised to be the cells of origin of glioma (Matarredona and Pastor, 2019). In this context, Ferguson et al. studied the role of LRIG1 in quiescence and activation of glioma stem cells (GSCs) making use of elegant strategies and gain and loss of function approaches. *In vitro* analyses pointed out the role of LRIG1 in cell proliferation and responsiveness to BMP signalling. Furthermore, transplantation of LRIG1-deficient GSCs in mice results in more aggressive tumours and decreased survival as compared with control GSCs, whereas LRIG1 overexpression elicited opposite effects, indicating that LRIG1 expression in GSCs enforces quiescence and represses tumour growth, paving the way to develop LRIG1-based anti-cancer therapies.

Exercise, diet, environmental enrichment and activities affecting cognition and learning exert a strong influence on neurogenesis (Ma et al., 2017; Gronska-Peski et al., 2021; Folsz et al., 2023; Melgar-Locatelli et al., 2023). Mazzitelli-Fuentes et al. describe for the first time that in adult teleosteans learning induces neurogenesis, adding insight to the relationship between these two processes across species, and showing that learning-induced neurogenesis is relevant in zebrafish specifically in two pallial regions: the caudal lateral pallium and the rostral medial pallium, involving NSCs proliferation and activity-dependent survival. In addition to those already mentioned, in the last decades, increasing evidence has pointed to social behaviour as an additional factor affecting adult neurogenesis. However, if and how do both phenomena influence each other is far from being well understood. In their review, Garcia-Gomez et al. nicely summarise the available evidence about bi-directional relationships between social behaviours and neurogenesis. The authors discuss how neurogenic changes induced either genetically or environmentally influence behaviour, and how social behaviours such as social living conditions, social enrichment, and social stress affect neurogenesis.

The regulation of the phenomenon of adult neurogenesis is complex and multifactorial, and affects all stages of the process, from NSC maintenance and self-perpetuation, differentiation into committed progeny, to the migration, survival and functional integration of new-born neurons. This comes as no surprise since being able to orchestrate the correct balance between quiescence and activation to ensure lifelong maintenance of an NSC pool, while producing the proper rate of differentiated progeny to meet the demands of an ever-adapting system as the brain, poses a formidable task. Surely, the list of NSC regulators, both intrinsic and contextual, will continue to expand in the following years as will the resolution and versatility of the techniques to study them.
